# Platinum cross-resistance after first-line PARPi maintenance in ovarian cancer: a review and proposed redefinition of platinum resistance

**DOI:** 10.3389/fphar.2026.1855919

**Published:** 2026-07-13

**Authors:** Rong Mei, Liang Song

**Affiliations:** 1 Hi-Tech Zone Hospital for Women and Children, West China Second University Hospital, Sichuan University, Chengdu, China; 2 Department of Medical Oncology, West China Second University Hospital, Sichuan University, Chengdu, China; 3 Key Laboratory of Birth Defects and Related Diseases of Women and Children (Sichuan University), Ministry of Education, Chengdu, China

**Keywords:** cross-resistance, first-line maintenance therapy, ovarian cancer, PARP inhibitors, platinum resistance, treatment-free interval

## Abstract

Poly(ADP-ribose) polymerase inhibitors (PARPis) as first-line maintenance therapy have significantly prolonged progression-free survival (PFS) in patients with advanced ovarian cancer and have become a standard of care. However, PARPis and platinum agents share DNA damage repair pathways, and preclinical and clinical studies suggest the existence of cross-resistance mechanisms. With the widespread use of first-line PARPi maintenance, whether it compromises the efficacy of subsequent platinum-based chemotherapy and whether the traditional definition of platinum resistance (treatment-free interval <6 months) needs to be revisited have emerged as urgent clinical issues. This review systematically summarizes the molecular mechanisms of cross-resistance, focuses on clinical evidence from the first-line maintenance setting (including real-world data and subgroup analyses of randomized controlled trials), and incorporates relevant updates from the National Comprehensive Cancer Network (NCCN) guidelines. Available evidence indicates that after first-line PARPi maintenance, patients have shorter subsequent time to next treatment (TTNT) and treatment-free interval (TFI). In the homologous recombination deficiency (HRD)-negative subgroup, overall survival point estimates are consistently >1 (PRIMA 1.09, PAOLA-1 1.19; 95% CIs crossed 1), consistent with the direction of the cross-resistance hypothesis. Based on these findings, we propose that the traditional definition of platinum resistance should be revised to incorporate prior PARPi exposure and molecular biomarkers. We also recommend caution when re-challenging with platinum after PARPi progression, prioritizing non-platinum regimens or clinical trials. Future prospective studies directly comparing different treatment strategies after first-line PARPi progression are urgently needed.

## 1 Introduction

Ovarian cancer is one of the deadliest gynecologic malignancies, with the majority of patients diagnosed at an advanced stage (III–IV). Although initial response rates to cytoreductive surgery combined with platinum-taxane chemotherapy are high, approximately 70% of patients relapse within 18 months after completing first-line therapy (Siegel et al., 2026). The introduction of poly(ADP-ribose) polymerase inhibitors (PARPis) has substantially changed the treatment landscape for advanced ovarian cancer. In patients who achieve a complete or partial response after first-line platinum-based chemotherapy, PARPi maintenance therapy significantly prolongs progression-free survival (PFS), as demonstrated in several randomized controlled trials (RCTs) including SOLO-1 (olaparib), PRIMA (niraparib), PAOLA-1 (olaparib plus bevacizumab), and ATHENA-MONO (rucaparib) (Gonzalez-Martin et al., 2019; Monk et al., 2022; Moore et al., 2018; Ray-Coquard et al., 2019).

However, with the widespread adoption of first-line PARPi maintenance, new clinical concerns have emerged. PARPis and platinum agents both act via DNA damage repair pathways, and preclinical studies have revealed overlapping resistance mechanisms, including restoration of homologous recombination (HR) function through *BRCA1/2* reversion mutations, enhanced replication fork protection, and upregulation of drug efflux pumps (Flynn and Ledermann, 2022; Summey and Uyar, 2022). Multiple retrospective studies and real-world data analyses have found that patients progressing after PARPi therapy may experience reduced efficacy of subsequent platinum-based chemotherapy, manifested as shorter treatment-free intervals (TFI), shorter time to next treatment (TTNT), and lower objective response rates (ORR) (Cecere et al., 2020; Frenel et al., 2022; Xu-Vuillard et al., 2024). This phenomenon has been termed “cross-resistance.”

Concurrently, the traditional definition of platinum resistance (relapse within 6 months after completing platinum-based chemotherapy) is based largely on clinical data from the 1980s–1990s (Markman and Hoskins, 1992). Whether this definition remains valid in the current era of widespread PARPi use warrants re-examination. The National Comprehensive Cancer Network (NCCN) 2026 guidelines explicitly state: “Definitions of platinum-sensitive and platinum-resistant disease represent a spectrum of disease; clinical judgment and flexibility should be utilized in determining treatment options” (NCCN, 2026). This official softening of the definition further highlights the urgency of re-evaluating platinum resistance in the PARPi era.

This review aims to systematically summarize the molecular mechanisms of cross-resistance between PARPis and platinum, integrate clinical evidence on the efficacy of subsequent chemotherapy after first-line PARPi maintenance (with emphasis on real-world studies and subgroup analyses of first-line RCTs), and explore potential revisions to the definition of platinum resistance and implications for clinical management. Understanding the pharmacological basis of this cross-resistance is essential for optimizing treatment sequencing and informing clinical pharmacology practice.

## 2 Mechanisms of cross-resistance between PARPis and platinum

Although PARPis and platinum agents have different primary targets, both induce tumor cell death by interfering with DNA damage repair pathways, and therefore they share multiple resistance mechanisms. It is important to note that the cross-resistance mechanisms described below are independent of the line of therapy: PARPi exposure at any line (first-line, second-line, or later) can induce resistance to subsequent platinum-based chemotherapy. Hence, from a biological perspective, first-line PARPi maintenance also carries a theoretical risk of inducing cross-resistance.

The key differences in DNA damage and repair pathways between platinum agents and PARPis are summarized in Table 1.

**TABLE 1:** Comparison of DNA damage features and main repair pathways between platinum agents and PARP inhibitors.

Feature	Platinum agents	PARP inhibitors
Type of DNA damage	Intrastrand crosslinks (predominant), interstrand crosslinks	PARP trapping, replication fork stalling, single-strand break accumulation
Primary repair pathways	Nucleotide excision repair (NER)	Base excision repair (BER), homologous recombination (HR)
Key repair proteins	ERCC1-XPF, XPA, XPG	PARP1, XRCC1, BRCA1/2
Main resistance mechanisms	NER upregulation, BRCA reversion (minor)	BRCA reversion, replication fork protection, drug efflux

Abbreviations: NER, nucleotide excision repair; BER, base excision repair; HR, homologous recombination; SSB, single-strand break; PARP, poly(ADP-ribose) polymerase.

### 2.1 Restoration of homologous recombination repair

In ovarian cancers harboring germline or somatic *BRCA1/2* mutations, the synthetic lethality of PARPis depends on loss of homologous recombination repair (HR) function. The most common mechanism of acquired resistance is secondary (reversion) mutations in *BRCA1/2* that restore the open reading frame and re-express functional BRCA protein, thereby re-establishing HR function (Kondrashova et al., 2017; Lin et al., 2019). In addition, loss of 53BP1, RIF1, or the shieldin complex can also bypass BRCA deficiency and restore HR, leading to resistance to both PARPis and platinum (Klotz and Wimberger, 2020). Among patients with platinum-resistant/refractory ovarian cancer, the detection rate of BRCA reversion mutations ranges from 13% to 18% (Summey and Uyar, 2022).

It is important to note that BRCA reversion-mediated HR restoration is a dominant mechanism of PARPi resistance but contributes less to platinum resistance, because platinum-DNA adducts are primarily repaired by the nucleotide excision repair (NER) pathway (Damia and Broggini, 2019). However, NER processing of platinum adducts generates DNA double-strand break intermediates that require HR for proper repair. Consequently, HR deficiency (e.g., BRCA mutation) enhances platinum sensitivity, whereas HR restoration via reversion mutations partially reduces platinum efficacy, albeit to a lesser extent than for PARPis. Moreover, upregulation of the NER pathway (e.g., ERCC1 overexpression) is a major mechanism of platinum resistance and has also been linked to PARPi resistance (Martin et al., 2008; Wu et al., 2023), indicating crosstalk between the two repair pathways.

### 2.2 Enhanced replication fork protection

Another important effect of PARPis is the induction of replication fork stalling and collapse, leading to double-strand DNA breaks. Resistant cells can evade this effect by enhancing replication fork stability, involving PARG loss, PI3K/AKT pathway activation, and alterations in the RAS/MAPK pathway (Gogola et al., 2018; McMullen et al., 2020). Recent studies have further identified that replication fork protection factors (e.g., CHTF18, RAD51) play critical roles in cross-resistance (Ray Chaudhuri et al., 2016; Kolinjivadi et al., 2017). These changes also reduce the cytotoxicity of platinum-induced DNA crosslinks.

### 2.3 Drug efflux and metabolic detoxification

Upregulation of ATP-binding cassette (ABC) transporter family members (e.g., P-glycoprotein, MRP2) increases efflux of both PARPis and platinum agents, reducing intracellular drug concentrations (Khan et al., 2021). Additionally, glutathione- and metallothionein-mediated detoxification of platinum also contributes to cross-resistance (Damia and Broggini, 2019).

### 2.4 Tumor microenvironment and metabolic reprogramming

The hypoxic microenvironment induces hypoxia-inducible factor (HIF) expression, which downregulates HR-related genes and promotes drug efflux and anti-apoptotic signaling (Chen et al., 2021). Tumor-associated macrophages (TAMs) polarized to an M2 phenotype secrete cytokines that activate NF-κB and STAT3 pathways, enhancing chemoresistance (Nowak and Klink, 2020). Cancer stem cells (CSCs), through high expression of ABC transporters and activation of Wnt/β-catenin and Notch pathways, serve as a reservoir of resistant cells (Pieterse et al., 2019).

### 2.5 Bidirectional cross-resistance: platinum prior to PARPi

Theoretically, cross-resistance should be bidirectional: resistance mechanisms induced by prior platinum exposure (e.g., NER upregulation, drug efflux) could also compromise subsequent PARPi efficacy. Preclinical studies support this notion (Gogola et al., 2018; Johnson et al., 2013). Clinically, Kondrashova et al. (2017) and Lin et al. (2019) reported that BRCA reversion mutations emerging after platinum therapy can also confer PARPi resistance. Recently, Kim et al. (2026) showed in a multicenter real-world study that the number of prior platinum lines negatively correlated with PFS on subsequent PARPi. However, direct evidence for the “platinum → PARPi” sequence remains limited and is confounded by selection bias, warranting further investigation.

In summary, multiple cross-resistance mechanisms can be induced upon PARPi exposure and are independent of the line of therapy. Therefore, first-line PARPi maintenance also carries a theoretical risk of inducing subsequent platinum resistance. Moreover, longer duration of PARPi exposure is associated with a higher probability of selecting resistant clones, suggesting that limiting the duration of PARPi maintenance or dynamically monitoring resistance mutations may help reduce the risk of cross-resistance.

**FIGURE 1:**
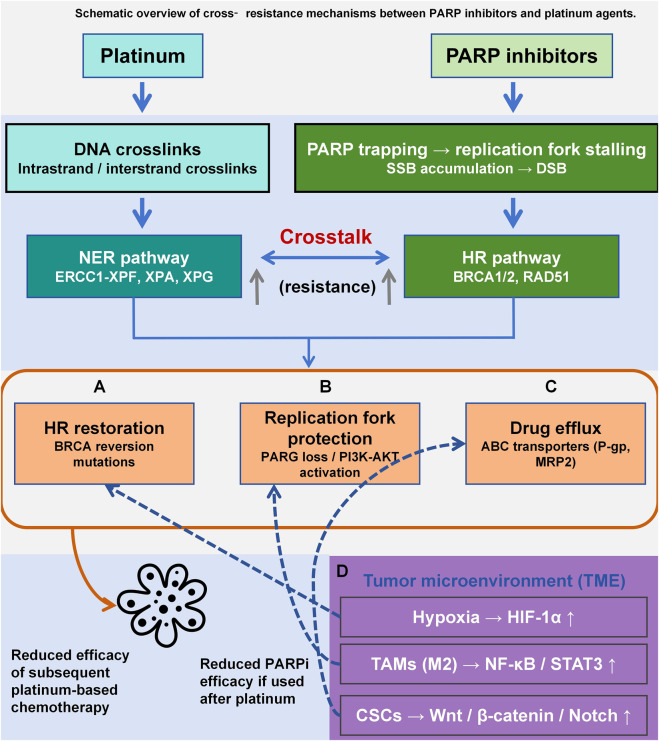
Schematic overview of cross-resistance mechanisms between PARPis and platinum agents. Platinum agents induce DNA intrastrand and interstrand crosslinks, which are primarily repaired by the nucleotide excision repair (NER) pathway (e.g., ERCC1-XPF). PARP inhibitors cause PARP trapping, replication fork stalling, and accumulation of single-strand breaks (SSBs) that convert to double-strand breaks (DSBs), requiring homologous recombination (HR) repair (e.g., BRCA1/2). The two pathways exhibit crosstalk, as NER processing of platinum adducts generates DSB intermediates that require HR. Three major cross-resistance mechanisms are highlighted: **(A)** BRCA reversion mutations restore HR function, conferring PARPi resistance and, to a lesser extent, platinum resistance; **(B)** enhanced replication fork protection (e.g., PARG loss, PI3K/AKT activation); stabilizes stalled forks, reducing cytotoxicity of both agents; and **(C)** upregulation of ABC transporters (e.g., P-glycoprotein, MRP2) increases efflux of both drugs. **(D)** The tumor microenvironment (hypoxia, tumor-associated macrophages, cancer stem cells) promotes all three resistance pathways via HIF-1α, NF-κB/STAT3, and Wnt/β-catenin/Notch signaling. The bidirectional dashed arrow between the two drugs indicates that prior exposure to either may induce cross-resistance to the other.

The major cross-resistance mechanisms are illustrated in Figure 1.

## 3 Clinical evidence on efficacy of subsequent chemotherapy after first-line PARPi maintenance

### 3.1 Real-world studies

Zucker et al. (2026) conducted a retrospective real-world study using the US Flatiron Health electronic health record database, including 3649 patients with ovarian cancer diagnosed between January 2015 and July 2023, of whom 621 (17%) received first-line PARPi maintenance (olaparib, niraparib, or rucaparib). The primary endpoints were time to next treatment (TTNT) and treatment-free interval (TFI).

Results showed that compared with patients who did not receive first-line PARPi, those who received first-line PARPi had significantly shorter TTNT2 (time from start of second-line to start of third-line treatment: 7.8 vs. 9.7 months) and TTNT3 (4.9 vs. 6.2 months), as well as shorter TFI2 (2.7 vs. 3.9 months) and TFI3 (1.1 vs. 1.7 months). In the BRCA-mutant subgroup, although first-line PARPi significantly prolonged TTNT1 (48 vs. 21 months), TTNT2 and TTNT3 were shorter than in BRCA-mutant patients who did not receive first-line PARPi (TTNT2: 7.6 vs. 17 months; TTNT3: 5.5 vs. 8.7 months) (Zucker et al., 2026).

Limitations of this study include unknown HRD status in 80% of patients, stratification only by BRCA status (BRCA-negative does not equal HRD-negative), selection bias (partially corrected for immortal time bias), and descriptive analysis without statistical testing. Nevertheless, this is the largest real-world study to date focusing on treatment outcomes after first-line PARPi maintenance, providing direct evidence that first-line PARPi exposure may be associated with shorter subsequent treatment intervals.

Zhang et al. (2024) provided supporting real-world data from an Asian population. This study included 102 patients who progressed after PARPi maintenance, of whom 45.1% had received first-line maintenance. Among 30 patients who received first-line PARPi maintenance followed by subsequent chemotherapy, the ORR was only 26.7% and median time to second progression (TTSP) was 7.1 months, substantially lower than historical controls without PARPi exposure in the ICON4 study (The Icon and ago collaborators, 2003) (ORR 54%–66%, PFS 10–13 months). These findings provide complementary evidence from an Asian cohort supporting reduced efficacy of subsequent chemotherapy after first-line PARPi exposure.

### 3.2 Indirect evidence from first-line RCTs

No prospective RCT has specifically evaluated the efficacy of subsequent chemotherapy after progression on first-line PARPi maintenance. However, overall survival (OS) results from published first-line maintenance RCTs, stratified by molecular subgroups, can provide indirect clues for the cross-resistance hypothesis. It is important to emphasize that this section only reports OS results for different molecular subgroups from each trial, without cross-subgroup extrapolation. All point estimates are reported together with their 95% confidence intervals (CIs), and statistical significance is clearly indicated.

SOLO-1 enrolled only patients with germline *BRCA1/2* mutations and advanced ovarian cancer, randomized to olaparib or placebo maintenance. At 7-year follow-up, median OS was not reached in the olaparib arm versus 75.2 months in the placebo arm (HR 0.55, 95% CI 0.40–0.76) (DiSilvestro et al., 2023). Although the trial did not report specific subsequent chemotherapy outcomes for patients who progressed on olaparib, the clear OS benefit suggests that in BRCA-mutant patients, the risk of cross-resistance does not outweigh the initial survival benefit of PARPi.

PRIMA enrolled an all-comer population (unrestricted by BRCA/HRD status) with advanced ovarian cancer, randomized to niraparib or placebo maintenance. Final OS analysis showed that in the HRD-positive subgroup (including BRCA-mutant patients), the OS HR was 0.95 (95% CI 0.70–1.29); in the HRD-negative subgroup, the OS HR was 1.09 (95% CI 0.84–1.42, p = 0.53) (Monk et al., 2024). The point estimate for the HRD-negative subgroup was >1, but the 95% CI crossed 1, indicating no statistical significance.

PAOLA-1 enrolled an all-comer population randomized to olaparib plus bevacizumab or bevacizumab plus placebo maintenance. Final OS results showed: BRCA-mutant subgroup HR 0.60 (95% CI 0.39–0.93); BRCA-negative/HRD-positive subgroup HR 0.71 (95% CI 0.45–1.13); HRD-negative subgroup HR 1.19 (95% CI 0.88–1.63, p = 0.29) (Ray-Coquard et al., 2023). Again, the point estimate for the HRD-negative subgroup was >1, but the 95% CI crossed 1.

A *post hoc* analysis of the PAOLA-1 trial (Harter et al., 2025) further evaluated the efficacy of first subsequent therapy (FST) in patients who progressed, using the time from FST to second subsequent therapy (FST→SST) as a measure of subsequent chemotherapy efficacy. The results showed that, regardless of HRD status, patients who progressed during olaparib maintenance had a significantly shorter FST→SST than those who progressed after the completion of olaparib maintenance. In the HRD-negative subgroup, the median FST→SST for progression on-treatment vs. after treatment was 6.0 months vs. 10.6 months (any chemotherapy as FST) and 6.6 months vs. 12.9 months (platinum-based chemotherapy as FST). Multivariable analysis confirmed that the timing of progression was an independent prognostic factor, distinct from platinum-free interval (PFI) and clinical risk (HR 0.65, 95% CI 0.50–0.84, P = 0.0011). This finding provides direct evidence that the duration of PARPi exposure and the timing of progression affect subsequent platinum efficacy, consistent with the cross-resistance hypothesis.

Integrated interpretation: In the HRD-negative subgroup, the OS point estimates from two independent trials (PRIMA and PAOLA-1) were both >1 (1.09 and 1.19, respectively). Although neither reached statistical significance, the consistency of direction suggests a potential risk signal, consistent with the cross-resistance hypothesis. In contrast, the clear OS benefit in the BRCA-mutant population indicates that this subgroup remains the greatest beneficiary of first-line PARPi maintenance.

The cumulative clinical and mechanistic evidence for cross-resistance raises a fundamental question: does the traditional binary definition of platinum resistance (based solely on TFI) remain adequate for patients who have received PARPi? Below we critically examine this definition and compare alternative proposals.

A summary of the design, results, and limitations of the above first-line studies is provided in Table 2.

**TABLE 2:** Summary of key studies evaluating subsequent chemotherapy efficacy after first-line PARPi maintenance.

Study	Design	Sample size	First-line PARPi (%)	Endpoint(s)	Key results	Limitations
Zucker et al. (2026)	Retrospective real-world (Flatiron)	3649	100%	TTNT, TFI	TTNT2: 7.8 vs. 9.7 months; TFI2: 2.7 vs. 3.9 months; BRCA mutants: shorter subsequent intervals	80% HRD unknown; no statistical tests; selection bias
Zhang et al. (2024)	Retrospective single-center	102	45.1%	ORR, TTSP	ORR 26.7%, TTSP 7.1 mo (first-line subgroup)	Small sample (n = 30 for first-line); single center
SOLO-1 (DiSilvestro et al., 2023)	Phase 3 RCT	391	100%	OS	BRCA mutant: OS HR 0.55 (95% CI 0.40–0.76)	No subsequent therapy data reported
PRIMA (Monk et al., 2024)	Phase 3 RCT	733	100%	OS	HRD-negative: point estimate 1.09 (95% CI 0.84–1.42)	Not statistically significant; no direct subsequent therapy analysis
PAOLA-1 (Ray-Coquard et al., 2023)	Phase 3 RCT	806	100%	OS	HRD-negative: point estimate 1.19 (95% CI 0.88–1.63)	Not statistically significant; no direct subsequent therapy analysis
PAOLA-1 *post hoc* (Harter et al., 2025)	Phase 3 RCT *post hoc*	544 (progressed)	100%	FST→SST	HRD-negative: progression on-treatment vs. after → median FST→SST 6.6 vs. 12.9 months (platinum-based); timing of progression independent prognostic factor (HR 0.65, 95% CI 0.50–0.84)	Post-hoc analysis; only progressed patients included

For real-world studies (Zucker, Zhang), p-values and CIs were not reported; only descriptive statistics are available.

*Abbreviations:* PARPi, poly(ADP-ribose) polymerase inhibitor; TTNT, time to next treatment; TFI, treatment-free interval; HRD, homologous recombination deficiency; ORR, objective response rate; TTSP, time to second progression; RCT, randomized controlled trial; OS, overall survival; HR, hazard ratio; CI, confidence interval; FST, first subsequent therapy; SST, second subsequent therapy.

### 3.3 Corroborative evidence from later-line/recurrent maintenance settings

Although patients in later-line/recurrent maintenance settings have multiple prior lines of therapy, higher tumor heterogeneity, and a higher incidence of BRCA reversion mutations, making them different from the first-line maintenance population, the cross-resistance mechanisms are biologically independent of line number. Thus, observations of reduced subsequent platinum efficacy after PARPi exposure in later-line populations can provide mechanistic corroboration and clinical caution for the risk of cross-resistance after first-line PARPi maintenance.

SOLO2 (olaparib maintenance for BRCA-mutant recurrent ovarian cancer) *post hoc* analysis showed that patients in the placebo arm who received subsequent chemotherapy after progression had a 7.3-month longer PFS than those in the olaparib arm (HR 1.42, 95% CI 0.89–2.27) (Frenel et al., 2022).

Cecere et al. (2020) reported a real-world study of 234 BRCA-mutant patients with recurrent ovarian cancer receiving olaparib maintenance. Among 66 patients who received subsequent chemotherapy after progression and were evaluable for response, ORR according to platinum-free interval (PFI) was 22.2% (12/54) for PFI >12 months, 11.1% (3/27) for PFI 6–12 months, and 9.5% (2/21) for PFI <6 months. These data indicate that even in traditionally platinum-sensitive patients (PFI >12 months), the ORR of subsequent platinum after PARPi exposure is only approximately 22%, substantially lower than the historical ORR of 54%–66% in platinum-sensitive recurrent patients without prior PARPi exposure (The Icon and ago collaborators, 2003).

Romeo et al. (2022) conducted a multicenter retrospective study involving 16 Spanish hospitals (n = 111), including patients with high-grade serous or endometrioid ovarian cancer who progressed after PARPi maintenance in the recurrent setting. Of these, 81 patients (73.0%) remained platinum-sensitive (PFI >6 months) after PARPi progression, and 74 received subsequent platinum-based chemotherapy. Analysis by BRCA status showed similar ORR between BRCA-mutant (n = 35) and wild-type (n = 39) patients (40.0% vs. 43.6%), but the rate of disease progression (as best response) was significantly higher in BRCA-mutant patients (45.7% vs. 17.9%, p = 0.004); median PFS (3.5 vs. 7.5 months, p = 0.03) and median OS (16.4 vs. 24.2 months, p = 0.036) were also significantly worse. This study suggests that after PARPi exposure, BRCA mutation may lose its value as a predictor of platinum sensitivity, and cross-resistance is particularly prominent in the BRCA-mutant population.

Recently, Kim et al. (2026) reported a multicenter retrospective study showing that prior PARPi exposure was associated with significantly shorter PFS on subsequent platinum-based chemotherapy, further corroborating the cross-resistance phenomenon.

Xu-Vuillard et al. (2024) conducted a multicenter retrospective study (n = 291) evaluating the efficacy of subsequent chemotherapy after PARPi exposure. Notably, only 14.1% of patients received first-line PARPi maintenance, while the vast majority (85.9%) received later-line PARPi, so the results mainly reflect the later-line population. Among patients with PFI >6 months, platinum-based chemotherapy was numerically superior to non-platinum chemotherapy (HR 0.68, 95% CI 0.46–1.01, P = 0.0547), but the difference did not reach statistical significance. Of note, this study found no effect of BRCA status on PFS (HR 1.19, P = 0.2972), which is not entirely consistent with the findings of Romeo et al. (2022) in the recurrent maintenance population. This discrepancy may be explained by a higher incidence of BRCA reversion mutations and other cross-resistance mechanisms in later-line populations, which attenuate the predictive value of BRCA status; it may also relate to differences in PFI distribution, duration of PARPi exposure, and subsequent treatment choices across studies. Overall, this study suggests that in later-line patients with PFI >6 months, platinum-based chemotherapy remains a viable option, but the predictive role of BRCA status requires further validation.

#### 3.3.1 Confounding factors

Outcomes in later-line settings are influenced not only by cross-resistance but also by declining performance status, cumulative toxicity, and increased tumor heterogeneity. To mitigate this confounding, we prioritized studies that performed multivariable analyses adjusting for performance status (e.g., Xu-Vuillard et al., 2024). The consistent signal across different study designs (RCT *post hoc*, real-world, and retrospective cohorts) supports a genuine biological effect beyond patient frailty.

The above studies mainly enrolled later-line or recurrent maintenance populations, and their results cannot be directly extrapolated to first-line patients across different molecular subgroups. However, the universality of cross-resistance mechanisms lends them corroborative value. In addition, regulatory agencies have also issued warnings about the long-term risks of PARPis: based on potential OS detriment observed in multiple RCTs, the US Food and Drug Administration narrowed or withdrew several PARPi indications in recurrent ovarian cancer during 2022–2023 (Shah et al., 2025), indirectly supporting the notion that PARPi exposure may adversely affect subsequent treatment outcomes.

## 4 Challenges and redefinition of platinum resistance

### 4.1 Origin and limitations of the traditional definition

The concept of platinum resistance dates back to the 1980s. Markman and Hoskins (1992) classified relapsed patients as platinum-sensitive (TFI ≥6 months) or platinum-resistant (TFI <6 months) based on the treatment-free interval (TFI, defined as time from last platinum dose to relapse). This binary classification has been widely adopted in subsequent clinical trials and clinical practice. However, Lindemann et al. (2018) challenged this dichotomy: among patients traditionally defined as platinum-resistant (TFI <6 months), the CA-125 response rate to platinum rechallenge was still significantly higher than to non-platinum regimens (51% vs. 21%, P < 0.001), and overall survival benefit was particularly evident in the subgroup with TFI 3–6 months. This indicates that the relationship between TFI and response to platinum rechallenge is not linear, and a simple binary classification may misguide treatment decisions.

Notably, international guidelines have begun to reflect on this definition. The NCCN 2026 guidelines (Version 1.2026) explicitly state in a footnote to the recurrence therapy pathway: “Definitions of platinum-sensitive and platinum-resistant disease represent a spectrum of disease; clinical judgment and flexibility should be utilized in determining treatment options” (NCCN, 2026). This official softening acknowledges the limitations of the traditional binary classification in the era of novel therapies and provides authoritative support for the redefinition proposed below.

### 4.2 Impact of PARPi exposure on the traditional definition

The widespread use of PARPis has further exposed the limitations of the traditional definition. First, the study by Zucker et al. (2026) showed that after first-line PARPi maintenance, TFI2 and TFI3 were significantly shortened (TFI2: 2.7 vs. 3.9 months; TFI3: 1.1 vs. 1.7 months), suggesting that PARPi may directly shorten subsequent treatment-free intervals. Second, even when TFI remains ≥6 months, the response to platinum rechallenge may be diminished due to cross-resistance. Therefore, in the PARPi era, relying solely on TFI to classify platinum sensitivity may overestimate platinum sensitivity, leading to inappropriate platinum rechallenge.

### 4.3 Proposed redefinition

Based on the above mechanistic and clinical evidence, we propose the following directions for redefining platinum resistance:

1. Add stratification factors: prior PARPi exposure (yes/no), duration of exposure (e.g., <12 months vs. ≥ 12 months), and time from PARPi progression to subsequent treatment.
2. Integrate molecular biomarkers: HRD status (HRD-positive vs. HRD-negative), BRCA mutation type (germline/somatic), CCNE1 amplification status, and dynamic monitoring of BRCA reversion mutations using liquid biopsy.
3. Dynamic assessment: Re-evaluate platinum sensitivity after each line of therapy, rather than relying solely on the TFI after first-line treatment.
4. Stratification example (see Table 3) to guide clinical decision-making and research design.

**TABLE 3:** Proposed reclassification of platinum sensitivity in patients with prior PARPi exposure.

Prior PARPi exposure	Molecular subgroup	TFI	Proposed new classification	Main rationale
Yes	HRD-negative	6–12 months	Platinum not preferred; prioritize non-platinum regimens	OS point estimates >1 (PRIMA 1.09; PAOLA-1 1.19), though not statistically significant; cross-resistance mechanisms; PAOLA-1 *post hoc*: progression on-treatment → shorter FST→SST (6.6 vs. 12.9 months for platinum-based)
Yes	HRD-negative	>12 months	Individualize; platinum may be considered with close monitoring (e.g., ctDNA for reversion mutations if applicable)	Limited evidence; long interval may retain partial platinum sensitivity, but caution advised
Yes	BRCA mutant	≥6 months	Platinum may be considered; consider baseline ctDNA testing for BRCA reversion mutations	Clear OS benefit in SOLO-1 (HR 0.55, 95% CI 0.40–0.76); however, Zucker et al. showed shorter TTNT after PARPi; reversion mutations predict resistance
Yes	HRD-positive (non-BRCA mutant)	≥6 months	Platinum may be considered, but evidence weaker than for BRCA mutants	PAOLA-1: HRD-positive overall population OS benefit (HR 0.63, 95% CI 0.45–0.85); NCCN recommends PARPi maintenance; however, post-PARPi platinum efficacy data limited
No	Any	By traditional definition	Unchanged	No PARPi interference

For patients with TFI <6 months, the traditional classification as platinum-resistant applies; prior PARPi, exposure does not change this classification. Such patients should receive non-platinum regimens regardless of molecular subgroup. The redefinition proposed in this table focuses on patients traditionally classified as platinum-sensitive (TFI ≥6 months). Clinical judgment should guide all treatment decisions.

Abbreviations: PARPi, poly(ADP-ribose) polymerase inhibitor; TFI, treatment-free interval; HRD, homologous recombination deficiency; OS, overall survival; FST, first subsequent therapy; SST, second subsequent therapy; TTNT, time to next treatment; ctDNA, circulating tumor DNA.

Clinical practice recommendation: For patients who progress after PARPi and are traditionally classified as platinum-sensitive (TFI ≥6 months), particularly those in the HRD-negative subgroup, platinum rechallenge should be approached with caution. Before platinum rechallenge, liquid biopsy to monitor for BRCA reversion mutations is advised, or non-platinum options (e.g., bevacizumab-containing regimens, non-platinum single agents) or clinical trials should be prioritized.

### 4.4 Comparison with alternative platinum resistance redefinitions

Several other groups have proposed refined or alternative definitions of platinum resistance:

- Lindemann et al. (2018) advocated treating TFI as a continuous variable rather than a binary cutoff. They found that even patients with TFI <6 months (traditionally platinum-resistant) had significantly higher CA-125 response rates to platinum rechallenge than to non-platinum regimens (51% vs. 21%, P < 0.001), and overall survival benefit was particularly evident in the TFI 3–6 months subgroup. This work challenged the fixed threshold but did not incorporate molecular biomarkers or PARPi exposure history.
- Lee et al. (2013) developed a nomogram integrating TFI, performance status, largest tumor size, CA-125, hemoglobin, and number of metastatic sites to predict overall survival in platinum-sensitive recurrent ovarian cancer. This tool improved prognostic accuracy but similarly did not account for PARPi exposure.
- ESMO-ESGO consensus (2024) recommends using HRD status to guide treatment in recurrent ovarian cancer but retains TFI as the primary stratification factor, without specific guidance for PARPi-pretreated patients.

Contribution of this review: Building on these prior frameworks, we propose a novel redefinition specifically tailored to PARPi-exposed patients, incorporating prior PARPi exposure, duration of exposure, HRD status, and dynamic ctDNA monitoring into a practical decision framework (Table 3).

## 5 Clinical implications and future research directions

### 5.1 Patient selection for first-line maintenance therapy

Current evidence suggests that the benefit-risk ratio of first-line PARPi maintenance differs significantly across molecular subgroups. For BRCA-mutant patients, the clear OS benefit supports continued use of PARPi maintenance. For HRD-positive/BRCA-wild-type patients, the OS benefit remains uncertain (PAOLA-1 HR 0.71 but not statistically significant), and the risk of subsequent cross-resistance should be discussed with patients. For HRD-negative patients, existing evidence (OS point estimates >1, real-world TTNT shortening, and the NCCN guideline statement that benefit is “minimal” (NCCN, 2026)) suggests limited benefit and potential risk from first-line PARPi maintenance, and such patients should be selected with extreme caution.

### 5.2 Treatment strategies after PARPi progression

Based on the risk of cross-resistance, blind platinum rechallenge should be avoided after PARPi progression. Recommended strategies include:

- Before platinum rechallenge, assess for BRCA reversion mutations or other evidence of HR restoration using ctDNA testing.
- Prioritize non-platinum options: bevacizumab plus chemotherapy (AURELIA regimen), non-platinum single agents (pegylated liposomal doxorubicin, weekly paclitaxel, topotecan), or clinical trials (e.g., ATR inhibitors, WEE1 inhibitors, immunotherapy combinations).
- For BRCA-mutant patients without evidence of reversion mutations, platinum rechallenge may be considered cautiously, but with close monitoring of efficacy and timing of resistance emergence.

**FIGURE 2:**
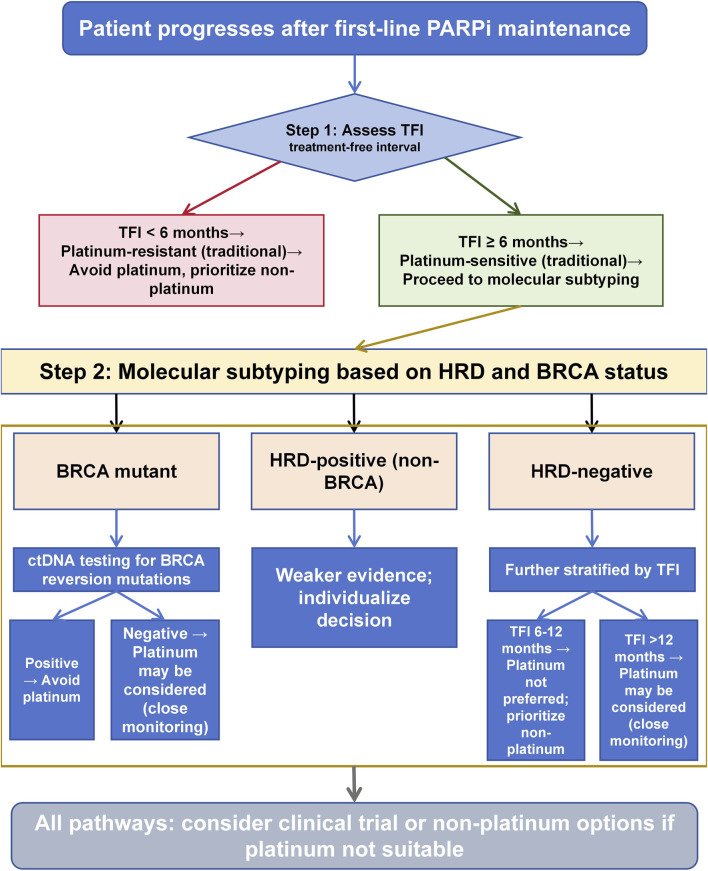
Clinical algorithm for platinum rechallenge after progression on first-line PARPi maintenance. The algorithm integrates treatment-free interval (TFI), molecular subgroup (BRCA mutant, HRD-positive non-BRCA, HRD-negative), and ctDNA monitoring for BRCA reversion mutations. For patients with TFI <6 months, traditional platinum resistance applies regardless of PARPi exposure; such patients should receive non-platinum regimens. For patients with TFI ≥6 months, molecular subtyping guides further decisions. In BRCA-mutant patients, ctDNA testing for reversion mutations is recommended; if negative, platinum may be considered with close monitoring. In HRD-positive non-BRCA patients, evidence for platinum benefit after PARPi is weaker, and decisions should be individualized. In HRD-negative patients, platinum is not preferred for TFI 6–12 months, but may be considered for TFI >12 months with close monitoring. All pathways ultimately consider clinical trials or non-platinum options.

A clinical algorithm for platinum rechallenge after first-line PARPi progression is provided in Figure 2.

### 5.3 Future research needs

1. Prospective randomized controlled trials: Directly compare platinum rechallenge versus non-platinum options (e.g., bevacizumab plus chemotherapy) after first-line PARPi progression, with endpoints including PFS2 and OS. (Estimated timeline: 5–7 years; feasibility: moderate, requiring large sample size and long follow-up.) Such trials should stratify patients by HRD status, prior PARPi exposure duration, and TFI length to account for the heterogeneity identified in real-world studies. Ideally, they would also include an adaptive design allowing for biomarker-driven treatment assignment based on ctDNA monitoring.
2. Biomarker studies: Validate the clinical utility of dynamic ctDNA monitoring for BRCA reversion mutations in predicting cross-resistance and guiding individualized therapy. Prospective validation of a clinically actionable threshold (e.g., variant allele frequency ≥1%) and the optimal monitoring interval (e.g., every 3–6 months) is needed. (Estimated timeline: 2–3 years; feasibility: high, can be performed within existing cohorts.) Additionally, exploration of other circulating biomarkers such as HRD-associated methylation patterns or exosomal microRNAs may further refine risk stratification.
3. Real-world evidence: Establish multicenter registries to systematically collect treatment patterns and outcomes after first-line PARPi progression, complementing the lack of RCT data and providing real-world support for definition revision. (Estimated timeline: 3–5 years; feasibility: moderate, requires multi-center collaboration.) Key data elements should include detailed PARPi exposure history (agent, duration, reason for discontinuation), TFI after PARPi, subsequent therapy type and response, and serial ctDNA results where available. These registries should aim for prospective data collection with standardized case report forms to minimize recall bias.
4. Mechanisms of acquired resistance after first-line PARPi: While preclinical studies have identified several cross-resistance mechanisms (e.g., BRCA reversion, replication fork stabilization), the frequency and clinical relevance of each mechanism in the first-line maintenance setting remain unclear. Longitudinal tumor and liquid biopsy sampling before PARPi initiation, at progression, and after subsequent therapy would help delineate the temporal evolution of resistance clones. (Estimated timeline: 3–5 years; feasibility: moderate, requires biobank support.) Single-cell sequencing and functional HRD assays could further elucidate whether resistance mechanisms differ between HRD-negative and HRD-positive tumors after PARPi exposure.
5. Cost-effectiveness and health economics: Given the high cost of PARPis and the potential for reduced subsequent platinum efficacy, formal cost-effectiveness analyses are warranted to determine the optimal duration of first-line PARPi maintenance from a health system perspective. (Estimated timeline: 1–2 years; feasibility: high, based on existing data modeling.) Such analyses should incorporate the downstream costs of salvage chemotherapy and the potential loss of platinum sensitivity, which may substantially affect the overall value proposition of first-line PARPi in different molecular subgroups.

## 6 Conclusion

Although first-line PARPi maintenance has significantly improved PFS in advanced ovarian cancer, accumulating evidence suggests that it may compromise the efficacy of subsequent platinum-based chemotherapy through cross-resistance mechanisms. Real-world studies (Zucker et al., 2026) have shown shortened TTNT and TFI after first-line PARPi exposure. In first-line RCTs, OS point estimates for the HRD-negative subgroup were consistently >1, consistent with the cross-resistance hypothesis. Mechanistic studies confirm that cross-resistance is independent of line of therapy. The traditional 6-month threshold for platinum resistance is no longer adequate in the PARPi era and requires revision to incorporate prior PARPi exposure and molecular biomarkers. In clinical practice, platinum rechallenge after PARPi progression should be approached with caution, especially in the HRD-negative subgroup. From a pharmacological perspective, the interaction between PARPis and platinum agents at the DNA repair pathway level creates a unique resistance profile that necessitates a paradigm shift in how we define platinum sensitivity. Future prospective studies directly comparing different treatment strategies after first-line PARPi progression are urgently needed, along with exploration of liquid biopsy-based individualized resistance monitoring.

## 7 Limitations

This review has several limitations:

1. Missing data in real-world studies: Zucker et al. (2026) had unknown HRD status in 80% of patients and stratified only by BRCA status (BRCA-negative does not equal HRD-negative), which may affect interpretation of HRD-negative subgroup evidence.
2. Lack of statistical tests: Real-world studies by Zucker and Zhang reported only descriptive statistics without between-group comparisons, limiting the strength of evidence.
3. Population extrapolation: The later-line/recurrent maintenance studies cited in Section 3.3 mainly enrolled BRCA-mutant or HRD-positive populations; their results cannot be directly extrapolated to HRD-negative patients or to the first-line maintenance setting.
4. Small subgroup sizes: Data for HRD-negative patients with TFI >12 months are extremely limited (only dozens of cases in RCT subgroup analyses); conclusions should be interpreted cautiously.
5. Potential confounding: Differences in later-line outcomes may be influenced by declining performance status, cumulative toxicity, and treatment selection bias; although we prioritized multivariable-adjusted studies, residual confounding cannot be excluded.
6. Lack of prospective validation of the proposed redefinition: The classification framework in Table 2 is based on existing evidence integration and has not been prospectively validated; its clinical utility and impact on patient outcomes require further study.
